# SEGF: A Novel Method for Gene Fusion Detection from Single-End Next-Generation Sequencing Data

**DOI:** 10.3390/genes9070331

**Published:** 2018-07-02

**Authors:** Hai Xu, Xiaojin Wu, Dawei Sun, Shijun Li, Siwen Zhang, Miao Teng, Jianlong Bu, Xizhe Zhang, Bo Meng, Weitao Wang, Geng Tian, Huixin Lin, Dawei Yuan, Jidong Lang, Shidong Xu

**Affiliations:** 1 Department of Thoracic Surgery, Harbin Medical University Cancer Hospital, Harbin 150049, China; xuhai18245159059@163.com (H.X.); dawei.sun@163.com (D.S.); bjl_1987@163.com (J.B.); 2 Department of Radiation Oncology, The First People’s Hospital of Xuzhou, Xuzhou 221002, China; Xiaojin.w@hotmail.com; 3 Department of Pathology, Chifeng Municiple Hospital, Chifeng 024000, China; cflishijun6588@sina.com; 4 Geneis Beijing Co., Ltd., Beijing 100102, China; zhangsw@geneis.cn (S.Z.); miao.teng@foxmail.com (M.T.); mengb@geneis.cn (B.M.); wangwt@geneis.cn (W.W.); tiang@geneis.cn (G.T.); linhx@geneis.cn (H.L.); yuandw-sci@geneis.cn (D.Y.); 5 Department of Anesthesiology, Chifeng Municiple Hospital, Chifeng 024000, China; zhangxizhe1965@sina.com

**Keywords:** fusion detection, single-end next-generation sequencing data, single-end gene fusion

## Abstract

With the development and application of next-generation sequencing (NGS) and target capture technology, the demand for an effective analysis method to accurately detect gene fusion from high-throughput data is growing. Hence, we developed a novel fusion gene analyzing method called single-end gene fusion (SEGF) by starting with single-end DNA-seq data. This approach takes raw sequencing data as input, and integrates the commonly used alignment approach basic local alignment search tool (BLAST) and short oligonucleotide analysis package (SOAP) with stringent passing filters to achieve successful fusion gene detection. To evaluate SEGF, we compared it with four other fusion gene discovery analysis methods by analyzing sequencing results of 23 standard DNA samples and DNA extracted from 286 lung cancer formalin fixed paraffin embedded (FFPE) samples. The results generated by SEGF indicated that it not only detected the fusion genes from standard samples and clinical samples, but also had the highest accuracy and sensitivity among the five compared methods. In addition, SEGF was capable of detecting complex gene fusion types from single-end NGS sequencing data compared with other methods. By using SEGF to acquire gene fusion information at DNA level, more useful information can be retrieved from the DNA panel or other DNA sequencing methods without generating RNA sequencing information to benefit clinical diagnosis or medication instruction. It was a timely and cost-effective measure with regard to research or diagnosis. Considering all the above, SEGF is a straightforward method without manipulating complicated arguments, providing a useful approach for the precise detection of gene fusion variation.

## 1. Introduction

Gene fusion occurs due to chromosomal breakage and rearrangement, and plays a critical role in oncogenesis. The oncogene fusion has the potential to increase protein expression, while fusions involving tumor suppressors tend to exhibit decreased expression. Recently, a number of gene fusions are being considered as diagnostic markers, for instance, the Philadelphia chromosome 9–22 translocation creates a fusion between *BCR* and *ABL1* genes in chronic myeloid leukemia. Also, rearrangement between chromosomes 11 and 22 is a characteristic of Ewing’s sarcoma, generating EWSR1–FLI1 fusion protein [1,2,3].

Traditional gene fusion detection methods, including fluorescent in situ hybridization (FISH) and PCR, lack scalability, as only a few genes can be evaluated simultaneously. Advances in next-generation sequencing (NGS) technology have provided a new approach to systematically identify genomic alterations. Compared to the traditional methods, NGS technology has many advantages, such as in sample throughput, cost-effectiveness, and detection sensitivities. Initially, many companies proceeded fusion gene detection tools for sequencing DNA and RNA to ensure accuracy, while this experimental design is both time- as well as money-consuming. Companies now prefer gene fusion analysis at the genomic level over the transcriptional level, because DNA capture enrichment technology is cost-effective and faster, and both single nucleotide variation (SNV) and gene fusion can be interrogated simultaneously. Hence, a proper method for precisely detecting gene fusion from DNA high-throughput sequencing is needed, and this subsequently increases the difficulty and complexity of data analysis. Currently, there are four predominant approaches for detecting structural variation:

1. De novo assembly: it detects structure variation in a more straightforward manner [4], but it is difficult to assemble the NGS short reads due to the influence of repetitive regions in the genome, as well as its high price.
2. Read-depth: it hypothesizes the read-depth of mapping reads matching the Poisson distribution, and then uses the actual read depth distribution to decide the structural variation. In terms of matched sample, structural variation is verified by comparing the deletion and duplication area between samples. For example, a study utilizes prior probability of single nucleotide combining hidden Markov model (HMM) to presume the type of structural variation [5]. Drawbacks of read-depth method are that its accuracy can be easily affected by amplification bias and sequencing bias.
3. Read-pair: it uses the feature of pair-end reads to measure the gap between their coordinates and direction. For reads with inconsistent span and direction, this method could speculate massive structural variation on account of large fragment insertion, deletion, translocation, and inversion. This method is restricted by the standard deviation of library fragment size, and the influence of software has an evident impact on the results. BreakDancer [6] is a widely-used tool for read-pair method.
4. Split-read: it discovers the breakpoint of structural variation through successfully mapped soft-clipped reads. The results are greatly affected by the repetitive sequences in genome and carefully consider the tool parameters. The commonly used split-read tools are Clipping REveals STructure (CREST) [7] and Fusion and Chromosomal Translocation Enumeration and Recovery Algorithm (FACTERA) [8].

We, inspired by the split-read method, developed a new approach to detect the target gene fusion variations using single-end reads, which was known as single-end gene fusion (SEGF). Single-end gene fusion directly uses raw sequencing data (FASTQ file) and integrates fast and simple alignment tools such as BLAST [9] and SOAP [10]. After filtering the analyzed data by stringent parameters, SEGF generates a list of fusion gene sequences. Compared with other methods that start with pair-end sequence data, SEGF method reduces sequencing costs and saves time significantly. Through comparing the results from PCR-fluorescence probing validation, it indicated that SEGF preceded other tools that utilize split-read method in terms of sensitivity and specificity.

## 2. Materials and Methods

### 2.1. Sample Collection

Five reference genomic DNAs (gDNAs) were used in this study, one was obtained from (Horizon Diagnostics™, Waterbeach, UK) and the other four were obtained from (Cobioer Biosciences Co., Ltd., Nanjing, China). HD753 is used as the reference standard from Horizon Diagnostics™, and it contains two fusion types, *ROS1–SLC34A2* (5.6%) and *RET–CCDC6* (5%). The original HD753 reference has two replicates, and its 3-diluted, 6-diluted, and 12-diluted samples have three replicates. ALK1 (*ALK–EML4* 28.30%), HP2 (*ALK–EML4* 20% and *ROS1–SLC34A2* 19%), LP (*ALK–EML4* 7.5% and *ROS1–SLC34A2* 6.7%), and ROS1 (*ROS1–SLC34A2* 22.53%), with reference standards, were obtained from Cobioer Biosciences Co., Ltd., and each sample has three replicates. As a result, 23 fusion positive samples from five gDNA reference standards after dilution and replication are obtained.

Informed consent was obtained from the 286 subjects from The Third Affiliated Hospital of Harbin Medical University. The 286 clinical samples we used were lung cancer formalin fixed paraffin embedded (FFPE) samples collected from January to July in 2017. These 286 clinical samples used 4 different capture panels, nonetheless, we did not separate them because we were going to check if the detection rate of *ALK-EML4* fusion from SEGF matches the clinical detection rate in lung cancer of Asian population. Among these, 16 samples are *ALK-EML4* fusion positive and 270 samples are *ALK-EML4* fusion negative according to PCR-fluorescence probing verification.

### 2.2. Gene Fusion Types Detected

Lung cancer patients are likely to carry *ALK-EML4*, *RET-CCDC6*, *ROS1-SLC34A2* fusion types [2]. Besides the frequent gene fusion types, we used a 14-gene set for gene fusion detection [11] as presented in Table 1. Theoretically, this approach allows all possible fusion types as specified in a matrix like Table 1.

### 2.3. Experimental Workflow

DNA for NGS-based gene fusion analysis was extracted using the GONOROAD Kit (Qiagen, Hilden, Germany) for FFPE tissue. DNA (200 ng) was used to build the library by using NEBNext Ultra II DNA library Prep Kit for Illumina (96 reactions) (NEB, Ipswich, MA, USA) Integrated DNA technologies (IDT, Skokie, IL, USA) customized probes were used for hybridization capture. Four different panels were used, and they all contained the 14 gene sets mentioned above. We used the 38 gene panel for all reference gDNA, 10 gene panel for 279 clinical samples, 143 gene panel for 6 clinical samples, 443 gene panel for 1 clinical sample and 38 gene panel for 4 abnormal samples. Quantification was performed with Library Quantification Kit—Illumina/Universal (Kapa Biosystems, Wilmington, MA, USA) on ABI 7500 Real Time PCR system (Applied Biosystems, Waltham, MA, USA). Quality control Agilent 2100 Bioanalyzer uses High Sensitivity DNA Kit for quality control (Agilent Technologies, Santa Clara, CA, USA). Next-generation sequencing -based fusion analysis was performed on Nextseq500 instrument according to the manufacturer’s instructions (Illumina, San Diego, CA, USA). With NextSeq500/550 High Output V2 kit, Illumina Nextseq500 (Illumina, San Diego, CA, USA) was used for DNA sequencing in 302 cycles, standing for Paired-End151bp.

PCR-fluorescence probing validation was performed with Detection Kit for EML4–ALK Fusion Gene Mutations (GBI, Beijing, China) and AmoyDx^®^ ALK Gene Fusions and ROS1 Gene Fusions Detection Kit (AmoyDx, Xiamen, China) according to the manufacturer’s instructions.

### 2.4. Single-End Gene Fusion Analysis Pipeline

**Figure 1:**
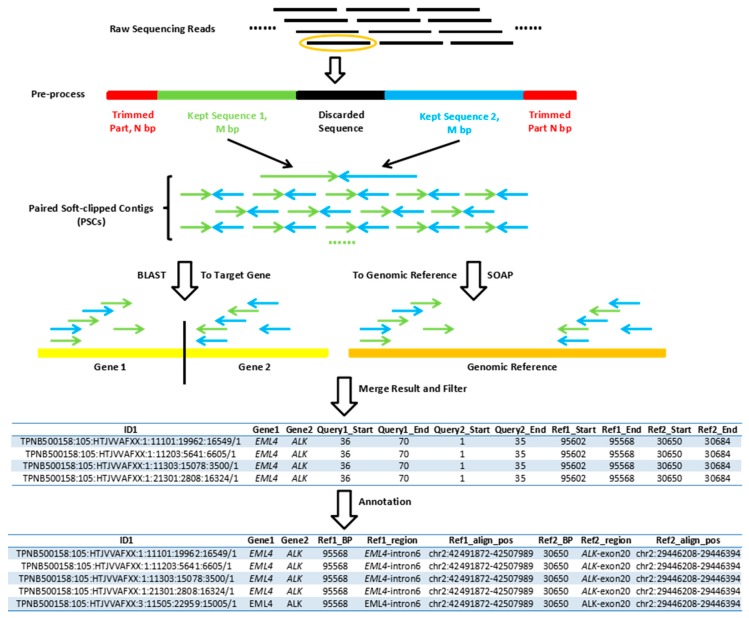
The scheme shows how the single-end gene fusion (SEGF) works. Firstly, there is pre-processing of raw sequencing data, including trimming of the first and last N bp (red part) and merging the first and last M bp of the remaining sequence as paired soft-clipped contigs (PSCs) (green and blue part); the last remaining part (black part) was discarded, and not used in the following analysis. Basic local alignment search tool (BLAST) and Short Oligonucleotide Analysis Package (SOAP) were used to align PSCs into target gene references (yellow part) and genomic references (orange part) separately, keeping the result unique and fully mapped to reduce the influence of genomic repetitive regions. The mutual sequences of the two filtered results are considered as fusion sequences. If the number of mutual reads was larger than three, then the sample was considered as fusion positive, otherwise, fusion negative.

Gene fusion involves the random combination of two genomic regions, reflecting the sequencing data, and a read containing gene fusion cannot map to the reference genome successfully. In the alignment process, reads containing a fusion breakpoint will be aligned to two homologous positions in the reference genome according to breakpoint. Considering the sequencing quality of the bases, the upstream and downstream of each read normally have a relatively high sequencing error rate, and paired-end data will double the account of sequencing errors and require more computing resources for calculation. Therefore, SEGF takes raw single-end sequencing data in zipped FASTQ format as an input file to perform analysis. Firstly, it trims 10 base pair (bp) from both sides of each read to remove the low-quality bases of the data, in order to increase the accuracy of the mapped results. Secondly, 35 bp sequences following the trimmed sequences from both ends were collected and merged together to generate a new sequence, which was defined as paired soft-clipped contigs (PSCs). Thirdly, the remaining sequences located between the two 35 bp sequences were discarded without being analyzed for further study. Since most fusion genes have an ambiguous breakpoint between the genes, it takes lot of computing time and brings many difficulties to define the sequence of the breakpoints, which was, however, not necessary for fusion gene discovery in the clinical study. Therefore, we designed SEGF to avoid the breakpoint sequences analysis, and retain the accurate gene information from 35 bp of both sides. A file in browser extensible data (BED) format, as shown in Section 2.2, should be provided to the tool, defining genes that are likely to fuse. Whole gene sequences of these genes would be extracted and merged together, then, this merged sequence was considered as the targeted gene sequence for alignment. Similarly, whole genome sequences containing all chromosomes were also needed for alignment, named as reference genome sequence. Target gene and reference genome sequences were indexed firstly. After that, the PSCs were analyzed by two methods. One is that we aligned PSCs to targeted gene sequences using basic local alignment search tool (BLAST) in the study, to get information regarding the possible fusion genes, then, the alignment result was filtered stringently, where no mismatches or gaps were allowed, and only 100% identical sequences were kept. The other analysis was that we aligned PSCs to a reference genome sequence, such as *GRCh37* (hg19), using Short Oligonucleotide Analysis Package (SOAP) analysis, and only kept unique mapped, no-mismatch and gapless sequences. The results derived from SOAP analysis provide us the information of whether or not the sequences of the reads are from other genomic sequencing areas, except the specific gene area. In addition, the result obtained from BLAST gives us the sequences that were accurately mapped to target gene sequence. Therefore, when we compared the two results, the sequences that can be found in both results had two features, which were uniquely mapped to reference genome sequence as well as precisely aligned to target gene sequence. In order to decide the status of each gene fusion type for each sample, only the detected sequences with more than two reads were considered as gene fusion positive samples, and the criterion is used in all clinical samples. Further annotations on those sequences were carried out and subsequently stored in tab-delimited text format, indicating the functional position of gene fusion sequences, such as intron or exon. The SEGF method was schematically depicted in Figure 1.

To evaluate the reliability of this approach, combination of two alignment tools and two gene fusion analysis methods were compared with SEGF. Burrows-wheeler aligner (BWA) is a well-known alignment tool, which is normally used in mapping steps. It has two alignment methods, ALN and MEM [12,13], which were designed for short reads and long reads respectively. FACTERA and CREST are two widely-used and accurate methods in structural variation analysis. These four tools were cross combined to four combinations, and the parameters for each tool are displayed as follows: BWA-ALN (-o:1 -e:50 -m:100,000 -t:4 -i:15 -q:10), BWA-MEM (default), FACTERA (default), CREST (-l = 151 bp), SEGF (trim_len:10 remain_len:35). These five alternatives were used to analyze the same data set to evaluate their sensitivity and specificity.

Availability and implementation: https://github.com/langjidong/SEGF.

## 3. Results

### 3.1. Assessment of Single-End Gene Fusion Detection of Multiple Gene Fusions in Reference Standards

To validate the feasibility of SEGF, we used SEGF to 23 fusion positive reference gDNA for assessing the effectiveness of gene fusion detection. These 23 standards contained *ALK–EML4*, *ROS1–SLC34A2*, and *RET–CCDC6* fusions, with a frequency of 7.5~28.3%, 0.47~5.6%, and 0.42~22.53%, separately. The average sequencing depth of 23 standards was 5191× ranging from 1879× to 12,626×. The detection result of cross comparison was represented in Table 2, wherein SEGF has a sensitivity of 95.65%, which was well above other methods. Among the 23 fusion positive standards, SEGF confirmed 22 samples, while FACTERA combining BWA-ALN and BWA-MEM verified 7 and 11 respectively, and CREST failed the assessment. It was worth mentioning that SEGF successfully validated three 12-diluted standard samples, whose *ROS1–SLC34A2* fusion frequency was decreased from 5% to 0.42%. Hence, SEGF was able to distinguish positive samples from negative ones with a fusion frequency as low as 0.4%. More details are displayed in the Supplementary Table S1.

### 3.2. Assessment of Single-End Gene Fusion Detecting ALK–EML4 Fusion in Clinical Samples

The viability of SEGF for fusion detection in reference standards was proven, with high sensitivity. Nonetheless, due to the difference in sample quality and fusion frequency between reference standards and clinical samples, a suitable method for the reference standard was not always applicable in the clinic. Hence, SEGF was applied to real clinical samples for evaluating its applicability. A total of 286 clinical samples were used, and 16 of these were *ALK–EML4* fusion positive, as confirmed by PCR-fluorescence probing, and 270 were negative. The average sequencing depth of these 286 samples was 6373×, ranging from 243× to 25,593×, and more information was represented in the Supplementary Table. FACTERA found no fusion positive samples, whether combined with BWA-ALN or BWA-MEM. On the other hand, BWA-ALN with CREST observed 3 positive samples, and BWA-MEM with CREST found 6 positives, with a positive detection rate of 1.05% and 2.1%, respectively. In the five approaches, SEGF detected 8 fusion positives with positive detection rate of 2.8%. Nevertheless, there were 8 PCR-verified positives that were not discovered by almost all of these approaches.

In 16 PCR-verified *ALK-EML4* positives, SEGF found eight, and then the experiment and analysis were repeated for the other 8 samples. Due to low gDNA input of clinical samples, four of eight samples do not have any tissues or library left, so they were considered as negatives, and consistent with the original result. For the rest of the 4 samples, we repeated the hybrid capture and sequencing of the libraries, and the average sequencing depth was 6451×, ranging from 5448× to 6897×. BWA-ALN + FACTERA, BWA-ALN + CREST, BWA-MEM + FACTERA and BWA-MEM + CREST discovered 1, 1, 2, 2 new positives in four re-analyzed samples separately, and on the other hand, SEGF found 3 new positives. Taken together, BWA-ALN + FACTERA, BWA-ALN + CREST, BWA-MEM + FACTERA, and BWA-MEM + CREST discovered one, four, two, eight positives, with sensitivity of 6.25%, 25%, 12.5%, and 50%, respectively. Nevertheless, SEGF observed 11 fusion positive samples with a sensitivity of 68.57%, and was superior compared to other approaches. The comparison of five approaches for *ALK–EML4* fusion detection of clinical samples was represented in Table 3.

## 4. Discussion

This study proposed a novel method called SEGF for gene fusion detection from NGS data, wherein single-end reads were used to predict the gene fusion at DNA level. Compared to the RNA-level gene fusion analysis, interrogation of DNA sequences enables us to obtain SNV information, gene fusion status, and other important biomarkers, such as microsatellite instability (MSI) status simultaneously, making DNA-seq more preferable. Single-end gene fusion has higher sensitivity than other methods in standard samples, as well as clinical samples. By contrast, the other four methods achieved extremely low detection rates in the two similar samples. Through inspecting the two standard samples named HP2 and LP, we observed an artificially edited sequence (GAAGTTCCTATTCCGAAGTTCCTATTCTTCAAATAGTATAGGAACTTC) inserted between *ALK* and *EML4* fusion, representing a kind of complex fusion type. The three-section-comprised fusion may be the reason for the failure in the detection by other methods. This type of sequence was unmappable, and discarded by successive gene fusion detection tools, because these tools tend to invoke soft-clipped sequences for fusion analysis. Since SEGF used two subsequences near the beginning and end of the raw sequence for analysis, it was able to avoid the detection failure on account of such complex fusion type. Therefore, SEGF had a higher sensitivity than other tools in terms of standard samples. Moreover, SEGF was able to observe fusion genes whose frequency in the whole standard data set was as low as 0.4%, which was calculated by the theoretical frequency divided by dilution ratio. On the other hand, among 286 clinical samples, SEGF found 11 *ALK–EML4* fusion positives, and the occurrence rate of 3.85%, which met the clinical positive rate of *ALK–EML4* in the Asian population [14,15,16]. Except for *ALK–EML4*, other fusion types, for instance, *RET–CCDC6* and *ROS1–SLC34A2*, were observed in the clinical samples, with higher sensitivity than other methods. As these results were not proved by PCR-fluorescence probing, they are not shown in the study.

Typical Illumina sequencing data has poor quality towards 3′ end of the DNA molecule, due to sequencing principles [17], and the quality fluctuates in the first 10 bases. Trimming of 10 bp at the start and end of sequences filters low quality bases and improves the alignment accuracy [18,19]. Generally, longer sequencing length contributes to more specific and precise alignment results and a better gene fusion detection rate, and it was also verified by our results from the SEGF pipeline analysis. By contrast, shorter sequences give more room for fusion breakpoint positions and makes it easier to analyze the gene fusion. With a certain length of the read length and strict mapping filters, longer sequence results in low mapping were possible for sequences consisting of gene fusions, while shorter sequences lead to multiple alignment positions and induces more noise. Due to the fact that the seed length of alignment tools ranged from 17 bp to 32 bp, we used 35 bp as default, but it can be adjusted by users according to the different sequencing read lengths and operation conditions.

The de novo assembly-based methods use pair-end, and require high-coverage sequencing data for gene fusion detection, but SEGF was not limited by these conditions. Read-pair based methods need pair-end sequencing data as well, and were highly affected by the range of fragment size. A read-depth based method was mainly used for copy number variation detection, and tends to be affected by amplification bias. Unlike other argument-dependent approaches, such as split-read based method, parameters are reduced as much as possible to prevent data loss in SEGF. It also makes great progress in saving the running time compared with other methodologies. For example, it took about 13 min to finish analyzing the standard sample named HP2-1 by using SEGF, compared with 25 to 28 min by using other methods. Nevertheless, more computer memory space was occupied when using SEGF, which spent 4.6 gigabits to analyze HP2-1 sample, while only 2 G was used in the other methods. This indicated that there was still space for SEGF optimization to make it more efficient for fusion gene discovery. We will focus on three aspects to improve the performance of SEGF. Firstly, remove duplications of PSC sequences before alignment, to reduce the number of input sequences for mapping step. Secondly, merge two mapping results in a multi-thread manner, for saving time. Thirdly, set more stringent filters for the two alignment results, to reduce the file size for the purpose of downstream merging as well as to reduce the memory used.

The successful detection of gene fusion is affected by many factors, including sample quality, the amount of fusion gene molecule existing in the sample DNA, etc. Predominantly, sample quality, and the amount of DNA input are vital for library construction, and the purity of tumor tissue may positively correlate to the amount of molecules containing gene fusions. The larger library fragment size makes it easier for SEGF to inspect the gene fusions as it increases the possibility of containing fusion breakpoint as well as benefits of alignment, because we can use longer sequences for precision alignment. If using liquid biopsy, it required a deeper sequence depth than tissue biopsy to retrieve as much information from cell-free DNA as possible. By finding the reason for detection inconsistency of 8 clinical samples, it was known that experiments greatly affect fusion discovery, including high-complexity steps, such as PCR and capture. The stabilization of experiments contributes a lot to gene fusion detection, as we can see from the inconsistency between the original experiment and repetitive experiment results. It remains crucial to ensure the experimental stabilization, due to the fact that there was only one experiment for clinical samples due to commercial reasons. Therefore, experimental steps should follow the standard operation protocols strictly, to have high repeatability and reliability.

## 5. Conclusions

To sum up, SEGF is capable for detecting complicated gene fusions, and not overly dependent on the choice of arguments. Single-end gene fusion saves money and time by using single-end sequencing data, but maintains high sensitivity and specificity. The application of SEGF is influenced by sample type, experiments, and sequencing quality, to a larger extent. Although the consistency of SEGF still needs improvement against the RNA level detection, SEGF is considered as a novel method for gene fusion detection in NGS.

## Figures and Tables

**Table 1:** Information of genes for gene fusion detection evaluation.

Chromosome	Start Position	End Position	Gene Symbol
Chr2	29415640	30144477	*ALK*
Chr2	42396490	42559688	*EML4*
Chr6	117609530	117747018	*ROS1*
Chr5	149781200	149792499	*CD74*
Chr4	25657435	25680368	*SLC34A2*
Chr10	43572517	43625797	*RET*
Chr10	61548506	61666414	*CCDC6*
Chr10	32297938	32345371	*KIF5B*
Chr20	43953929	43977064	*SDC4*
Chr6	159186773	159239340	*EZR*
Chr1	154134289	154164611	*TPM3*
Chr12	59265937	59314319	*LRIG3*
Chr6	117881433	117923705	*GOPC*
ChrX	133594175	133634698	*HPRT1*

**Table 2:** Comparison of gene fusion detection results for reference standards.

Method	TP	TN	FP	FN	Sensitivity	Specificity
BWA-ALN + FACTERA	7	0	0	16	30.43%	-
BWA-ALN + CREST	0	0	0	23	0.00%	-
BWA-MEM + FACTERA	11	0	0	12	47.83%	-
BWA-MEM + CREST	0	0	0	23	0.00%	-
SEGF	22	0	0	1	95.65%	-

TP: true positive, TN: true negative, FP: false positive, FN: false negative, BWA: Burrows-wheeler aligner; FACTERA: Fusion and Chromosomal Translocation Enumeration and Recovery Algorithm CREST: Clipping REveals STructure.

**Table 3:** Comparison of *ALK–EML4* fusion detection results for clinical samples.

Method	Sample	TP Rate	TP	TN	FP	FN	Sensitivity	Specificity
BWA-ALN + FACTERA	286	0.35%	1	270	0	15	6.25%	100.00%
BWA-ALN + CREST	286	1.40%	4	270	0	12	25.00%	100.00%
BWA-MEM + FACTERA	286	0.70%	2	270	0	14	12.50%	100.00%
BWA-MEM + CREST	286	2.80%	8	270	0	8	50.00%	100.00%
SEGF	286	3.85%	11	270	0	5	68.75%	100.00%

## Supplementary Materials

The following are available online at http://www.mdpi.com/2073-4425/9/7/331/s1, Table S1: Sequencing data statistics and gene fusion detection result for standard samples.
